# Relationship between compassion fatigue, medical narrative ability, and retention intention among nurses: a cross-sectional multi-center study

**DOI:** 10.3389/frhs.2026.1718055

**Published:** 2026-03-20

**Authors:** Fengju Wu, Yanjia Li, Jue Wu, Limei Zhang, Guoting Ma, Shaoping Wu, Yanli Hu

**Affiliations:** 1 Nursing Department, Sichuan Taikang Hospital, Chengdu, Sichuan, China; 2 Science and Education Department, People’s Hospital of Mingshan District, Ya’an City, Sichuan, China; 3 School of Nursing, Chengdu Medical College, Chengdu, Sichuan, China; 4 Department of Anesthesiology, Gansu Provincial Hospital, Lanzhou, Gansu, China; 5 Radiology Department, Sichuan Taikang Hospital, Chengdu, Sichuan, China; 6 School of Nursing, Guangzhou Medical University, Guangzhou, Guangdong, China

**Keywords:** compassion fatigue, medical narrative ability, nurse, retention intention, study

## Abstract

**Background:**

In recent years, due to the increase in care demands caused by population aging and the rise in nurse-patient conflicts, the retention intention of nurses has become an urgent global issue, as high turnover rates pose significant challenges to healthcare systems around the world. Therefore, this study will explore nurses’ retention intention and the associated factors.

**Methods:**

This multi-center cross-sectional study recruited 1,831 nurses from eight hospitals across China between January and February 2024 through convenience sampling. Data collection was conducted using online surveys. The questionnaire was divided into four sections: the sociodemographic questionnaire, the Chinese Questionnaire for Nurse Intention to Remain Employed (C-QNIRE), the Medical Narrative Ability Scale (MNAS), and the Chinese version of Compassion Fatigue Short Scale (C-CF-Short Scale). We utilized descriptive statistics, normality test, one-way ANOVA, *t*-tests, Pearson correlation analysis, and multiple linear regression analysis.

**Results:**

The results of this study showed that the total score of nurses' retention intention was 22.79 ± 3.71. The Multiple linear regression results showed that compassion fatigue (*β* = −0.334, *p* < 0.001), medical narrative ability (*β* = 0.250, *p* < 0.001), age (46–60 years) (*β* = 0.143, *p* < 0.001), age (41–45 years) (*β* = 0.121, *p* < 0.001), age (36–40 years) (*β* = 0.107, *p* < 0.001), age (31–35 years) (*β* = 0.093, *p* < 0.001), working week (>50 h) (*β* = −0.096, *p* < 0.05), working week (46–50 h) (*β* = −0.071, *p* < 0.05), working week (40–45 h) (*β* = −0.070, *p* < 0.05), how many night shifts a month (>8) (*β* = −0.062, *p* < 0.05) were the main associated factors of nurses' retention intention, explaining 27.0% of the total variation.

**Conclusion:**

This study indicated that nurses demonstrate a moderate retention intention. Nursing administrators and educators should address this issue by proactively introducing strategies to reduce compassion fatigue and improve narrative medicine ability among nursing staff. Such initiatives can positively impact nurses’ retention intention, thereby reducing the turnover of skilled nursing personnel. This is essential for effective management of nursing human resources and enhancing the overall quality of nursing care.

## Introduction

As global healthcare systems continue to advance, nurses remain indispensable in delivering medical care. However, recent years have witnessed a concerning increase in nurse turnover rates worldwide, particularly in high-stress environments, where the retention intention declined (1). A decrease in retention intention implies greater job mobility and a higher likelihood of nurses leaving their positions. This trend not only jeopardizes the quality and stability of nursing services but also increases recruitment and training costs for hospitals, potentially disrupting the smooth operation of the entire healthcare system (2). In China, this phenomenon is particularly striking. With the increasing aging of the population, the impact of COVID-19 on the global economy, and the implementation of Diagnosis Related Groups (DRG)-based medical insurance payment reform in China, nurses' retention intention has further decreased (3). Additionally, layoffs and even bankruptcies at some medical institutions have exacerbated the trend. Therefore, it is crucial to conduct a thorough analysis of the current state of nurses' retention intention and its associated factors within nursing management research. Such studies can improve nursing management practices and provide a solid basis for healthcare institutions to formulate effective human resource strategies.

The retention intention of nurses reflects their desire and probability to continue working in those positions (4). The factors that influence this retention intention are diverse and intricate. Studies show that nurses with strong retention intention tend to remain in the nursing field for longer durations, demonstrate higher job involvement, and positively contribute to the quality of nursing care (5). On the other hand, nurses with weaker retention intention are more prone to leaving their jobs, which can negatively impact healthcare organizations and patient outcomes (6). A thorough review of existing literature indicates that the primary drivers of nurses' retention intention include aspects such as the work environment, compensation, opportunities for career advancement, and personal psychological and emotional well-being (7). In addition, some studies have pointed out that new nurses may experience greater stress due to their lack of experience, while senior nurses may accumulate fatigue due to long-term exposure (8). Age, length of service, and professional title are often related to experience, responsibility, and career development stage, which affect stress perception, coping resources, and intention to leave (2). Therefore, we speculate that these sociodemographic variables may also have some relationship with nurses’ retention intention. Therefore, we speculate that these sociodemographic variables such as age, frequent overtime work, and frequent staying up late may also have a certain relationship with nurses' retention intention.

Prolonged high-pressure work environments and emotional investment expose nurses to varying levels of emotional exhaustion, making compassion fatigue a critical concern for occupational mental health. Compassion fatigue is marked by the depletion of emotional, mental, and physical resources due to continuous exposure to the distress and trauma experienced by others (9). Studies show that this condition not only negatively impacts the mental well-being of nurses but can also diminish work efficiency, job satisfaction, and the overall quality of nursing care (10). According to Charles Figley's Compassion Fatigue Theory, the main drivers often include the demanding work environment, patient suffering, and the emotional needs of patients (11). In intensive nursing roles, while offering emotional support to patients, nurses must also cope with their own emotional strain and occupational stress. This dual burden is frequently the primary factor leading to compassion fatigue, which subsequently affects job satisfaction (12). Additionally, research by Lee et al. (13) highlights a significant link between job burnout and retention intention among nurses, and also indicating a positive correlation between compassion fatigue and job burnout, potentially accelerating turnover rates (14, 15). Therefore, it is reasonable to infer that compassion fatigue may also be related to the nurses' retention intention.

In recent years, the medical narrative ability of nurses has increasingly attracted academic interest as a critical element in their interaction and communication with patients. Medical narrative ability involves healthcare providers’ ability to understand and interpret patients’ accounts of their illnesses, convey the underlying meanings of these narratives accurately, empathize with patients, and advocate for their best interests (16). Drawing from Hochschild's Emotional Labor Theory, which posits that service professions demand employees to control and modulate their emotional expressions to fulfill job requirements (17). Medical narrative ability can be seen as a type of emotional labor. It requires nurses to manage and express emotions effectively, listen attentively to patients’ experiences, and cultivate trust and empathy during care (18). However, nurses often encounter heightened demands for emotional labor, especially when this labor lacks adequate support, potentially leading to greater job burnout and decreased retention intention. Research also suggests that effective medical narratives can strengthen the emotional bond between nurses and patients, enhance patient treatment experiences, and increase patient satisfaction (19). On the other hand, insufficient medical narrative skills may result in suboptimal communication and increased professional stress for nurses (20). While some studies have investigated the impact of medical narrative ability on nurses’ occupational health, fewer have explored its connection with retention intention. Considering the prevalent issues of job burnout and stress among nurses, often associated with lower retention rates (13). Medical narrative ability provides a valuable form of emotional support. It enables nurses to better handle emotional challenges at work. We propose that improving medical narrative ability could help alleviate these challenges, and improve nurses' retention intention.

Nurses' retention intention is shaped by an intricate combination of various factors. Although some studies have explored the relevant factors of nurses' retention intention, the impact on the specific situation, regional differences and cultural background of Chinese nurses needs to be further studied. Therefore, this study aims to investigate the current situation of retention intention among Chinese nurses through a multi-center cross-sectional survey, explore the correlation between retention intention, compassion fatigue, and medical narrative ability, and propose strategies to improve nurse's retention intention from a new perspective. This approach seeks to provide theoretical support and practical guidance for enhancing the stability of the nursing team, optimizing the allocation of nursing resources, and improving the quality of nursing services. Based on the above background, we propose the following research hypotheses for this study:

Hypothesis 1: The compassion fatigue of nurses is negatively correlated with retention intention.

Hypothesis 2: The medical narrative ability of nurses is positively correlated with retention intention.

Hypothesis 3: The age of nurses, working week, and the number of night shifts per month may all affect nurses' retention intention.

## Methods

### Design and participants

This study employed convenience sampling to conduct an online questionnaire survey among 1,831 nurses from 8 hospitals across the country from January to February 2024. The inclusion criteria were as follows: (i) age ≥18 years old; (ii) obtain the professional qualification registration of nurses; (iii) voluntary participation in this study; (iv) the working time is more than 6 months. The exclusion criteria are as follows: (i) nurses who were not on duty during the survey period; (ii) participants who withdrew voluntarily during the study period. The Strengthening the Reporting of Observational Studies in Epidemiology (STROBE) guidelines were followed. The G-power 3.1 program was used to determine the minimum sample size required for the study with *α* set at 0.05, effect size (*f*^2^) at 0.15, power (1−*β*) at 0.95, and the number of predictors at 14. It turned out that 129 participants were needed. Considering a 20% attrition rate, this study required at least 154 participants. However, the actual effective sample size of this study was 1,831 cases, which far exceeded the theoretical requirements.

### Measures

This study used four measures to collect data: the sociodemographic questionnaire (gender, age, education level, marital status, professional title, department, working experience, income, how many night shifts a month, have you experienced a major traumatic event in the past year, working week), the Chinese Questionnaire for Nurse Intention to Remain Employed (C-QNIRE), the Medical Narrative Ability Scale (MNAS), and the Chinese version of Compassion Fatigue Short Scale (C-CF- Short Scale).

Nurses' retention intention was measured using the C-QNIRE, which was translated by (21). The C-QNIRE is a one-dimensional scale consisting of 6 items. Each item is scored on a 1–5 Likert scale, with items 2, 3, and 6 reverse-scored. The C-QNIRE ranges from 6 to 30 points. The higher the score, the higher the retention intention. In this study, the Cronbach's alpha coefficient of the C-QNIRE was 0.749. The content validity correlation coefficients of the items in C-QNIRE range from 0.590 to 0.810.

Nurses' medical narrative ability was measured using the MNAS, which was developed by (16). The MNAS consisted of 27 items categorized into three domains: pay attention and listen, understanding and response, reflection and representation. The items are rated on a 7-point Likert Scale ranging from 1 (“completely inconsistent”) to 7 (“completely consistent”). In order to better present the results, the total score of the scale ranges from 27 to 189 points, and the higher the score, the stronger the medical narrative ability of the individual. In this study, the Cronbach's alpha coefficient for the MNAS was 0.967 and the content validity was 0.890.

The Compassion Fatigue Scale (CFS) was developed by Adams et al. (22), in 2006. The Chinese version of Compassion Fatigue Short Scale (C-CF-Short Scale) was revised by Lou (23). The scale consists of two dimensions, secondary traumatic stress (5 items) and burnout (8 items), with a total of 13 items. It uses a Likert 10-point scale from 1 (“never”) to 10 (“very often”). The total score of this scale ranges from 13 to 130 points, and the higher the score, the higher the level of individual empathy fatigue. Cronbach's alpha coefficient of the C-CF- Short Scale was 0.955 in this study. The content validity correlation coefficients of the items in C-CF- Short Scale range from 0.830 to 0.870.

### Data collection and recruitment

We distributed an online questionnaire to 1,840 eligible and voluntarily active nurses in eight hospitals nationwide. The online questionnaire was created using a survey tool called “So Jump” and delivered through the social software WeChat to the research heads of each hospital. Subsequently, researchers at each hospital selected suitable participants based on predetermined criteria and provided them with information about the purpose of the questionnaire as well as instructions for completion within a designated time frame. To prevent duplicate responses, only one submission per WeChat account was allowed for completing the questions ensuring comprehensive participation. In total, we disseminated 1,840 questionnaires resulting in receiving 1,831 valid responses with an effective recovery rate of 99.5%. Furthermore, this study employed a convenience sampling method, where eligible on-the-job nurses were recruited by the heads of each collaborating hospital. Although this method has limitations in terms of representativeness, its advantage lies in the ability to efficiently and feasibly complete data collection across multiple research centers nationwide within a short period of time. This is the practical choice for the operational aspect of this large-scale cross-sectional study.

### Ethics approval and consent to participate

Ethical approval for this study was obtained from the Ethics Committee of Sichuan Taikang Hospital (No. SCTK-IRB-2024-030). This research was conducted in strict accordance with the Helsinki Declaration. All the data collected were anonymized and strictly kept confidential. Informed consent was obtained from all participants.

### Statistical analyses

Data analysis was conducted using IBM SPSS 27.0. The normality of distributions for nurses’ retention intention, medical narrative ability, and compassion fatigue was evaluated using Kolmogorov–Smirnov (KS) test. Descriptive statistics were applied to provide an overview of the participants' demographic characteristics and the scores of the main study variables. To assess differences in retention intention according to participant characteristics, one-way ANOVA and independent samples *t*-tests were employed. Pearson correlation analysis was used to explore the relationships among key variables. Before conducting the multiple linear regression analysis, we tested the basic assumptions of the model. The linear relationship between variables was initially judged by observing the scatter plot of standardized residuals and standardized predicted values; homoscedasticity was evaluated by checking whether the distribution of residuals in the scatter plot with predicted values was random and uniform; multicollinearity was assessed using the variance inflation factor (VIF), and the VIF of all independent variables was <10, indicating no serious multicollinearity problem. After the test, the data of this study met the basic assumptions of multiple linear regression. Additionally, multiple linear regression analysis was carried out to determine significant related factors of retention intention. Statistical significance was set at *p* < 0.05 for two-tailed tests.

## Results

### Description of the participants

Before data analysis, we standardized the main variables. In addition, the Harman single-factor test method was used to test the common method bias of the original data. The results showed that there were 8 factors with eigenvalues greater than 1, and the explanation rate of the first factor was 38.22%, which was lower than the critical value of 40%, indicating that there was no common method bias. A total of 1,831 valid questionnaires were collected in this study. Respondents were predominantly female (*n* = 1,783, 97.4%), and the majority of participants (*n* = 1,308, 71.4%) had at least a bachelor's degree or above. In addition, only a small percentage of participants (*n* = 483, 26.4%) were single, divorce or others. About half of the nurses earned less than 5,000 RMB per month (*n* = 805, 44%). The remaining general demographic information is shown in Table 1.

**Table 1:** Personal characteristics and single factor analysis of retention intention.

Variables	*N* (%)	*M* ± SD (score)	*t*/*F*	*p*	Cohen's *d/η*^2^
Gender			2.923	0.004	3.700
Female	1,783 (97.4)	22.83 ± 3.68			
Male	48 (2.6)	21.25 ± 4.38			
Age (years)			17.888	<0.001	0.047
18–25	210 (11.5)	21.70 ± 3.60			
26–30	601 (32.8	22.33 ± 3.79			
31–35	500 (27.3)	22.71 ± 3.56			
36–40	259 (14.1)	23.21 ± 3.59			
41–45	124 (6.8)	24.18 ± 3.32			
46–60	137 (7.5)	24.72 ± 3.48			
Marital status			19.000	0.088	0.020
Married	1,348 (73.6)	23.06 ± 3.70			
Single	410 (22.4)	21.81 ± 3.60			
Divorce or others	73 (4.0)	23.37 ± 3.45			
Education level			−0.465	0.642	3.708
College	523 (28.6)	22.73 ± 3.71			
University or above	1,308 (71.4)	22.82 ± 3.71			
Professional title			14.048	<0.001	0.023
Nurse	267 (14.6)	22.21 ± 3.63			
Senior nurse	738 (40.3)	22.41 ± 3.67			
Nurse-in-charge	701 (38.3)	23.15 ± 3.72			
Associate chief nurse or above	125 (6.8)	24.28 ± 3.48			
Department			1.808	0.094	0.006
Internal medicine	688 (37.6)	22.54 ± 3.80			
Surgical	432 (23.6)	22.75 ± 3.71			
Pediatric	86 (4.7)	23.34 ± 3.12			
Obstetrics and gynecology	115 (6.3)	23.33 ± 3.19			
Outpatient	83 (4.5)	23.43 ± 3.79			
Emergency	87 (4.8)	22.55 ± 3.72			
Others	340 (18.6)	22.93 ± 3.76			
Working experience (years)			19.510	<0.001	0.041
<3	212 (11.6)	21.77 ± 3.66			
3–5	319 (17.4)	22.38 ± 3.67			
6–10	426 (23.3)	22.23 ± 3.75			
11–15	521 (28.5)	23.04 ± 3.62			
>15	353 (19.3)	24.08 ± 3.47			
Income (RMB/month)			10.083	<0.001	0.016
<3,000	106 (5.8)	21.32 ± 4.01			
3,000–5,000	699 (38.2)	22.64 ± 3.66			
5,001–10,000	978 (53.4)	22.98 ± 3.69			
>10,000	48 (2.6)	24.44 ± 2.87			
How many night shifts a month			17.287	<0.001	0.028
0	406 (22.2)	23.66 ± 3.63			
1–4	569 (31.1)	23.02 ± 3.58			
5–8	455 (24.8)	22.51 ± 3.66			
>8	401 (21.9)	21.90 ± 3.79			
Have you experienced a major traumatic event in the past year			2.728	0.006	3.701
No	1,703 (93.0)	22.85 ± 3.70			
Yes	128 (7.0)	21.93 ± 3.78			
Working week (hours)			14.568	<0.001	0.023
<40	195 (10.6)	23.59 ± 3.32			
40–45	1,038 (56.7)	23.03 ± 3.60			
46–50	402 (22.0)	22.43 ± 3.94			
>50	196 (10.7)	21.46 ± 3.75			

### Single factor analysis of retention intention

Kolmogorov–Smirnov (KS) test showed that the data for compassion fatigue, medical narrative ability, and retention intention were approximately normally distributed (*p* > 0.05). Therefore, mean and standard deviations were used to describe the states of these variables. The differences in the nurses' retention intention with the gender, age, professional title, working experience, income, how many night shifts a month, have you experienced a major traumatic event in the past year, and working week (*p* < 0.05) (Table 1).

### Descriptive statistics of compassion fatigue, medical narrative ability, and retention intention

In this study, the total mean score of nurses' compassion fatigue, medical narrative ability, and retention intention were 48.03 ± 27.27, 154.48 ± 22.93, 22.79 ± 3.71 (Table 2).

**Table 2:** Descriptive analyses of compassion fatigue, medical narrative ability, and retention intention.

Variables	Min	Max	*M* ± SD (score)
Compassion fatigue			
Secondary traumatic stress	5	50	16.60 ± 10.94
Burnout	8	80	31.43 ± 17.94
Total score	13	130	48.03 ± 27.27
Medical narrative ability			
Pay attention and listen	19	63	50.06 ± 7.41
Understanding and response	12	84	69.48 ± 11.34
Reflection and representation	6	42	34.94 ± 5.66
Total score	38	189	154.48 ± 22.93
Retention intention			
Total score	6	30	22.79 ± 3.71

### Correlation between nurses' compassion fatigue, medical narrative ability, and retention intention

The results of the Pearson correlation indicated that compassion fatigue was negatively correlated with medical narrative ability (*r* = −0.238, *p* < 0.01) and retention intention (*r* = −0.411, *p* < 0.01). The medical narrative ability was positively correlated with retention intention (*r* = 0.344, *p* < 0.01) (Table 3).

**Table 3:** Correlation coefficients of the main study variables.

Variables	Compassion fatigue	Medical narrative ability	Retention intention
Compassion fatigue	1		
Medical narrative ability	−0.238**	1	
Retention intention	−0.411**	0.344**	1

** *p* < 0.01.

### Multiple linear regression analysis of nurse' retention intention

In this study, nurses' retention intention score was used as the dependent variable, and the statistically significant variables in univariate analysis and correlation analysis were taken as independent variables; we conducted multivariate linear regression analysis. The independent variable assignment methods were either original value input or dummy variable assignment (age, professional title, professional title, department, working experience, income, number of night shifts per month, working week). The results showed that compassion fatigue, medical narrative ability, age, working week, and how many night shifts a month entered the regression equation (*p* < 0.05) (Table 4).

**Table 4:** The multivariate linear regression analysis of nurse’ retention intention.

Variables	*B*	SE	*β*	*t*	*p*	95% CI	VIF	Tolerance
Constant	18.680	0.653	-	28.592	<0.001	17.399 to 19.960	-	
Compassion fatigue	−0.045	0.003	−0.334	−16.066	<0.001	−0.051 to −0.040	1.081	0.925
Medical narrative ability	0.040	0.003	0.250	12.115	<0.001	0.034 to 0.047	1.068	0.936
Age (46–60 years)	2.008	0.372	0.143	5.392	<0.001	1.278 to 2.738	1.752	0.571
Age (41–45 years)	1.788	0.370	0.121	4.836	<0.001	1.064 to 2.513	1.576	0.635
Age (36–40 years)	1.135	0.297	0.107	3.818	<0.001	0.552 to 1.718	1.960	0.510
Age (31–35 years)	0.776	0.262	0.093	2.967	0.003	0.263 to 1.289	2.479	0.403
Working week (>50 h)	−1.148	0.332	−0.096	−3.458	0.001	−1.798 to −0.497	1.921	0.521
Working week (46–50 h)	−0.633	0.281	−0.071	−2.256	0.024	−1.183 to −0.083	2.464	0.406
Working week (40–45 h)	−0.522	0.249	−0.070	−2.100	0.036	−1.010 to −0.035	2.773	0.361
How many night shifts a month (>8)	−0.554	0.251	−0.062	−2.210	0.027	−1.046 to −0.063	1.964	0.509

*R*^2^ = 0.275, Adjusted *R*^2^ = 0.270, *F* = 53.124, *p* < 0.001.

## Discussion

The results of this study indicated that nurses' retention intention was at a moderate level, consistent with the findings of Liu et al. (4). Following the significant economic impact of the global coronavirus pandemic, China has also experienced a general reduction in nurses' compensation (24). Moreover, some hospitals in China have cut expenses, increasing the risk of layoffs and bankruptcy, which has inadvertently heightened work pressure on nurses and influenced their retention intention (8). With the intensification of global aging, the demand for medical care among the elderly in China has risen. Long-term high-intensity work, emotionally demanding labor, and relatively low salaries are likely key factors contributing to nurses' reduced retention intention (25). The decline in nurses' retention intention not only exacerbates nurse turnover but also threatens the quality of nursing services and the stability of the healthcare system. Therefore, it is recommended that nursing managers enhance retention intention by improving salary and welfare benefits, offering career development and promotion opportunities, and ensuring rational allocation of human resources (26).

Our research findings indicate that the higher the compassion fatigue of nurses is, the lower their retention intention will be. This finding underscores the substantial impact of compassion fatigue, a specific manifestation of occupational fatigue, on nursing professionals. Research indicates that nurses experience high levels of emotional labor, particularly in high-pressure departments such as emergency and ICU units, leading to elevated levels of compassion fatigue (27). Intense and close emotional interactions with critically ill or terminally ill patients can intensify emotional consumption, resulting in emotional exhaustion, job burnout, anxiety, depression, and other mental health issues, thereby diminishing career satisfaction and retention intention (28). Therefore, hospital and nursing managers should be aware of the long-term effects of compassion fatigue and invite relevant psychotherapists to implement interventions such as cognitive behavioral therapy (CBT) and mindfulness-based stress reduction (MBSR) (29, 30). Additionally, providing adequate organizational support, reasonable scheduling, and workload adjustments can help alleviate compassion fatigue and enhance nurses' retention intention.

Our research findings indicate that the higher the medical narrative ability of nurses is, the stronger their retention intention in their positions will be. Specifically, as nurses' medical narrative ability increases, so does their retention intention. This ability not only enhances emotional interaction with patients but also strengthens nurses' identification with their professional roles (31). Effective communication through storytelling allows nurses to build stronger trust relationships with patients, better understand patient emotions and needs, and thereby increase their sense of identity and engagement in nursing work (18). This finding aligns with existing research that identifies medical narrative ability as a critical factor in enhancing nurses' emotional support and professional value (18). By improving this skill, nurses can achieve greater job satisfaction, harmonize nurse-patient relationships, and reduce work-related stress, ultimately boosting their retention intention. Therefore, it is recommended that hospitals and nursing managers regularly implement narrative medicine training programs while providing comprehensive emotional and psychological support to help nurses enhance their communication skills, empathize with patients, manage emotional demands, and improve overall job satisfaction and retention intention (32, 33).

This study reveals that compared with nurses aged 18–25, those aged 31–60 show a stronger retention intention, while younger nurses have a lower retention intention. Research indicates that older nurses, having experienced multiple career stages, typically possess extensive work experience and a strong sense of professional identity, enabling them to maintain high retention intention despite work pressures (34). In contrast, younger nurses in the early stages of their careers are more prone to job burnout within a relatively short period due to uncertainties in career development paths, excessive occupational pressure, unclear career prospects, and dissatisfaction with salary and benefits (35). Additionally, young nurses often face instability in personal and family life, leading to higher career mobility and greater susceptibility to changes in the external environment and career opportunities, which further reduces their retention intention (36). Therefore, it is recommended that hospital managers provide older nurses with more challenging positions, management opportunities, or academic research spaces to sustain their work motivation and loyalty (37). For younger nurses, offering comprehensive vocational training, promotion opportunities, and career planning can effectively enhance their retention intention (38, 39).

Our research reveals that compared with nurses working 40 h or less per week, those working more than 40 h per week have a lower retention intention. This finding highlights the adverse impact of frequent overtime on the mental health and retention intention of nurses, providing an important basis for improving working conditions in the nursing industry and increasing the retention intention (40). In China, the shortage of nursing human resources has led to widespread overtime work among nurses. Research indicates that nurses working longer hours experience higher levels of occupational fatigue and emotional strain, which diminishes their enthusiasm for nursing and reduces their willingness to remain in the profession (41). The excessive workload, particularly for frontline clinical nurses, not only compromises their quality of life and personal relationships but also poses significant health risks, ultimately affecting career development and retention intention (42). Therefore, ensuring a reasonable distribution of working hours to promote work-life balance is essential for enhancing nurses’ retention intention (43).

This study reveals that nurses who work more than eight night shifts per month have a lower retention intention compared to those who do not work night shifts. Prolonged nocturnal work disrupts circadian rhythms, leading to diminished sleep quality and compromised immune function, among other health issues (44). Additionally, night shifts are typically characterized by heavy workloads and high psychological stress. Night shift nurses often bear greater responsibilities independently, which can negatively impact their family life and social interactions. This heightened emotional burden not only exacerbates psychological pressure but also diminishes job satisfaction and enthusiasm, thereby affecting retention intention (45). Therefore, it is recommended that nursing managers optimize the scheduling of night shifts to prevent clustering of consecutive night shifts and ensure adequate rest periods between shifts (46, 47). Furthermore, enhancing working conditions for night shift nurses, such as providing comfortable rest areas and appropriate financial incentives, can effectively alleviate the associated stress and fatigue (46, 47).

## Limitations

This research explored the connections among compassion fatigue, medical narrative ability, and nurses' retention intention. Utilizing a multi-center cross-sectional survey approach, this study bolstered the reliability and applicability of its outcomes, thereby offering a theoretical basis for formulating specific intervention strategies aimed at enhancing nurses' retention intention. Despite its significant contributions, the study is not without limitations. Firstly, there are methodological limitations. In terms of sampling methods, this study adopted convenience sampling, which may lead to sample selection bias and affect the representativeness and extrapolation of the results. Future research can adopt stratified random sampling and other methods to improve the coverage of the sample to the target population. Secondly, in terms of measurement tools, the data were mainly collected through self-administered questionnaires, which may be affected by social expectation effects, common method bias, and recall bias, reducing the measurement accuracy. Future research can combine behavioral observation, objective indicators, or multi-time-point tracking data to improve the reliability and validity. In terms of sample representativeness, the subjects of this study were all from Chinese hospitals, and the conclusions may be influenced by the specific medical system, occupational culture, and management model in China. The cross-cultural and cross-regional applicability of the conclusions still need to be further verified. Second conceptual and theoretical limitations. This study mainly focused on the statistical relationships between variables, and the path relationships between variables were still based on cross-sectional data, which cannot strictly infer causal relationships. Future research can be verified by combining longitudinal designs and actual resignation data. Third background and practical application limitations. The conclusions of this study originated from a specific period and medical environment, and did not fully consider the moderating effects of situational factors such as policy changes, hospital levels, and department differences. In addition, when translating research findings into specific intervention measures, there may be practical challenges such as resource constraints, implementation feasibility, and acceptance by medical staff. Therefore, when applying the results of this study for management decisions, adjustments and verifications should be made based on local actual conditions. To further improve the research quality, future studies can conduct cross-cultural comparative research, introduce multi-level modeling methods, adopt mixed research designs, and conduct intervention-based empirical research. This will enable a more comprehensive and dynamic revelation of the relationships between variables, and enhance the theoretical value and practical significance of the results. The statistical analysis of this study did not include a formal diagnosis of outliers (such as through indicators like the Cook distance). Although we used robust standard errors analysis to enhance the reliability of the results, future research could incorporate a more systematic process for outlier detection and handling in the analysis to further verify the robustness of the model.

## Conclusion

This research indicated that nurses demonstrated a moderate retention intention. The study found that compassion fatigue had an inverse relationship with retention intention, whereas medical narrative ability showed a positive correlation. This research holds clear value both in theory and practice. In terms of theoretical contribution, the study innovatively introduces the concept of narrative ability from medical humanities into the research on nurses' professional psychology, revealing that medical narrative ability serves as an internal mechanism for alleviating compassion fatigue and enhancing retention intention. This goes beyond the previous focus on work conditions and psychological exhaustion, providing a new framework that integrates emotions and cognition, as well as individual and care contexts, to understand nurses' professional experiences and retention motivations. It also fills the knowledge gap in existing literature regarding how “positive psychological resources” specifically act on the resilience of the nursing profession. Secondly, in terms of practical and policy implications, the study directly addresses the urgent issues of global nursing workforce shortage and high turnover rate, and provides empirical evidence for interventions at different levels. For instance, medical institutions can systematically conduct narrative medicine training and reflection practice groups to enhance nurses' medical narrative ability, thereby buffering the negative impact of compassion fatigue. Nursing education institutions should strengthen the cultivation of medical narrative ability in their curricula and internships, helping nursing students establish early abilities in emotional regulation and meaning construction. Policy makers can base their decisions on the conclusions of this study and promote the inclusion of narrative support mechanisms and occupational mental health programs in nursing human resource planning. Through institutional design, they can promote the stability and quality of the nursing team. Therefore, this research not only expands the intersectional field between nursing professional psychology and medical humanities, but also provides specific paths for implementing targeted and evidence-driven intervention measures, which is conducive to enhancing nurses' professional happiness and retention intention, and ultimately ensuring the continuity and high quality of medical services.

## Data Availability

The original contributions presented in the study are included in the article/Supplementary Material, further inquiries can be directed to the corresponding authors.
